# Footcare in the dust…providing podiatry across the Kimberley

**DOI:** 10.1186/1757-1146-4-S1-P14

**Published:** 2011-05-20

**Authors:** Brydie M Donnelly, Lesley Newcombe

**Affiliations:** 1 Boab Health Services, Broome, Western Australia 6725, Australia

## 

The Kimberley is a region in the north of WA which covers 423 517 square km’s (approximately twice the size of Victoria and three times that of England!). Although the Kimberley is a large geographical area the population is estimated at only 38 000 people who live in many remote locations right across the region. Thus providing reasonable healthcare services in this area often requires clients and care providers to travel long distances. Two of the main contributors to morbidity and mortality in the region are type II diabetes, and chronic kidney disease. Because of the high rate of foot complications associated with these disorders there are many people who require podiatric care, however, podiatric services in the Kimberley are limited and only three podiatrists cover this vast area. The podiatry team work out of many locations and with a variety of services to provide clinical podiatric care. The podiatrists also offer staff in-service and up-skilling, as well as community group education sessions on a range of topics and conditions at the many locations that they visit. The podiatrists travel to over 15 remote communities and five towns, to provide services and the tyranny of distance means that they often drive for long hours to provide these services. All equipment needed must be taken with them as there is no podiatry clinics already established. The focus of the service is on indigenous clients with chronic disease such as diabetes, and due to the limitations of the service, clients must meet an eligibility criteria. Over 2,600 occasions of service were provided in 2009 with 64% of clients being indigenous. The podiatrists are mainly involved with the assessment and management of foot problems related to chronic disease such as diabetes. The full range of podiatric care is provided, including wound management, general foot care, biomechanical assessment, minor nail surgery and the provision of footwear via Community Aids and Equipment Program or via donated footwear.

